# *LPL*, *FNDC5* and *PPARγ* gene polymorphisms related to body composition parameters and lipid metabolic profile in adolescents from Southern Italy

**DOI:** 10.1186/s12967-022-03314-w

**Published:** 2022-03-03

**Authors:** Benedetta Perrone, Paola Ruffo, Samanta Zelasco, Cinzia Giordano, Catia Morelli, Ines Barone, Stefania Catalano, Sebastiano Andò, Diego Sisci, Giovanni Tripepi, Corrado Mammì, Daniela Bonofiglio, Francesca Luisa Conforti

**Affiliations:** 1 Medical Genetics Laboratory, Department of Pharmacy and Health and Nutritional Sciences, University of Calabria, Rende, CS Italy; 2 Olive Growing and Olive Oil Industry Research Centre, Agricultural Research Council, 87036 Rende, CS Italy; 3 Department of Pharmacy, Health and Nutritional Sciences, Via P Bucci, 87036 Rende, CS Italy; 4 Centro Sanitario, University of Calabria, Via P Bucci, 87036 Rende, CS Italy; 5 Institute of Clinical Physiology of Reggio Calabria, IFC-CNR, Reggio Calabria, Italy; 6 Medical Genetics Unit, Great Metropolitan Hospital BMM, Reggio Calabria, Italy

**Keywords:** Health, SNP, LDL, Lipids, Anthropometric parameters

## Abstract

**Background:**

Plasma lipid profile and anthropometric variables are known to be under strong genetic control and the identification of genetic variants associated with bioclinical parameters is of considerable public health importance. In this study, a young cohort of healthy individuals was genotyped for genes related to health and pathological conditions, to analyze the association of single nucleotide polymorphisms (SNPs) with different bioclinical parameters, adherence to the Mediterranean Diet (MD) and physical activity, studying the role of lifestyle and body composition parameters on biochemical metabolic profile.

**Methods:**

Association analysis of single variants in the genes of lipoprotein lipase (*LPL*), fibronectin type III domain containing protein 5 (*FNDC5*), and peroxisome proliferator-activated receptor-gamma (*PPARγ*) and haplotype analyses were performed.

**Results:**

Multiple (n = 14) common variants in the three genes demonstrated a significant effect on plasma lipoprotein-lipid levels and/or on biochemical parameters in our sample. Specifically, SNPs were related to lipid metabolism (rs3866471, rs4922115, rs11570892, rs248, rs316, rs1059507, rs1801282) or glycemic profile (rs3208305) or anthropometric parameters (rs3480, rs726344, rs1570569) for a total of 26 significant associations (P < 0.01 and/or P < 0.05) and two haplotypes, for the first time, were strongly associated with lipid and body composition parameters. Interestingly, we identified twenty-four new variants not previously described in the literature and a novel significant association between rs80143795 and body composition.

**Conclusions:**

In this study we confirm the association between these SNPs on lipid metabolism and body parameters also in a young cohort, indicating the important role of these genetic factors as determinants of health.

**Supplementary Information:**

The online version contains supplementary material available at 10.1186/s12967-022-03314-w.

## Background

Increasing scientific evidence has shown that predisposition to a particular health condition is not due to the modification of a single gene but to the variation of multiple genes located in numerous parts, or loci, of the chromosomes that can be affected by the interaction with the surrounding environment. Among the environmental factors, proper nutrition and regular physical activity can improve the quality of life contributing to a good state of health [1].

Therefore, much of the current biomedical research is based on the expectation that understanding the genetic contribution to health status will revolutionize the diagnosis, treatment, and prevention of diseases [2].

DNA sequence variation analysis is becoming a prominently important source of information for identifying genes involved in both normal biological and disease processes, such as development, aging, and reproduction. In this context, information about genetic variation is critical for understanding how genes work or malfunction and genetic and functional variation correlate.

In particular, individual lipid profiles appear to be strictly determined by lifestyle (smoking, diet, and physical activity). Family studies suggested that in many populations about half the variation in these traits is genetically determined and the concentrations of LDL cholesterol, HDL cholesterol, and triglycerides are strongly influenced by individuals’ genetic makeup. Indeed, genetic variants represent a substantial fraction of individual variation in lipid concentrations [3]. However, several articles focusing on the role of genetic variation in anthropometric and body composition parameters have revealed genetic markers that influence the functional response of the human body to regular physical activities [4, 5], the legacy of these parameters remains unclear.

Lipoprotein lipase (*LPL*), fibronectin type III domain containing protein 5 (*FNDC5*—precursor of irisin), and peroxisome proliferator-activated receptor gamma (*PPARγ*) are well-known candidate genes since the key roles of their products in lipid and energy metabolism and/or their involvement in the pathogenesis of various complications of dyslipidemia has been extensively documented [6–9]. In particular, *LPL* encodes the lipoprotein lipase enzyme which hydrolyzes triglycerides and functions as a ligand/bridge factor for receptor-mediated absorption of lipoproteins; *FNDC5* encodes a protein that is released by muscle cells during exercise and regulates energy metabolism by converting white to brown fat, contributing to muscle adipose tissue cross-talk; *PPARγ* encodes for a member of PPAR family of nuclear receptors which is an important adipogenic regulator and a modulator of intracellular insulin-signaling events [10–12].

Several traditional SNP genotyping methods based on single traditional PCR or PCR real-time with fluorescent probes are commonly used in genetic research. However, the decreasing cost coupled with rapid progress in Next Generation Sequencing (NGS) and related bioinformatic computing resources have facilitated large-scale discovery of SNPs, genotyping thousands of SNPs in one run. This study aimed at identifying genetic polymorphisms in *LPL, FNDC5,* and *PPARγ*, using high-performance technologies combined with the evaluation of biochemical and anthropometric/body composition parameters, in healthy Italian adolescents enrolled in the Mediterranean Diet and Sport research program.

## Methods

### Subjects

The study was carried out between December 2018 and January 2021 at the laboratory of Medical Genetics, Department of Pharmacy and Health and Nutritional Sciences, University of Calabria. A sample of 92 (44 females and 48 males) genetically unrelated, healthy subjects of Caucasian descent (aged 14–17 years), was recruited from students at the public high school “Istituto Istruzione Superiore”—Castrolibero (CS). Biochemical blood tests were performed to complement the evaluation of the individual’s overall status. Measurements of anthropometric characteristics, body composition parameters, and physical activity levels of participants enrolled within the Italian project DIMENU (Dieta Mediterranea and Nuoto) were previously described [13–15]. The questionnaire of Adherence to the Mediterranean Diet (MD), KIDMED Test, was used to assess the adherence to the MD in the study population with the score ranging from 0 to 12 points. In addition, the intensity of physical activity levels were defined based on the WHO recommendations (https://www.who.int/news-room/fact-sheets/detail/physical-activity) and adolescents were grouped in physical inactivity (Group A; < 3 METs), moderate physical activity (Group B; 3–6 METs), and vigorous-intensity physical activity (Group C; > 6 METs). Table 1 reports demographic and lifestyle characteristics of the participants. Considering that sample integrity is critical in genomic experiments and several variables (e.g. collection and storage) may influence it, 77 DNA samples underwent targeted genetic sequencing. (Table 1). Parents of all participants provided written informed consent and the study was conducted according to the guidelines established in the Declaration of Helsinki and approved by the Ethics Committee of the University of Calabria, Italy (# 5727/2018).

**Table 1:** Characteristics of study participants

Characteristics	N = 77
Male %	44.16
Age, years	15.76 ± 1.09
BMI (Kg/m^2^)	23.02 ± 3.71
WHR (cm)	0.78 ± 0.06
TBW (%)	27.86 ± 5.13
BMR (Kcal)	1559.27 ± 188.01
BCM (Kg)	27.80 ± 6.43
FFM (%)	77.45 ± 11.42
FM (Kg)	13.1 ± 7.07
Total cholesterol (mg/dL)	150.08 ± 27.92
HDL (mg/dL)	50.22 ± 11.65
LDL (mg/dL)	85.39 ± 22.56
Triglycerides (mg/dL)	75.86 ± 61.95
Glucose (mg/dL) Insulin (µIU/mL)	79.77 ± 6.86 10.21 ± 13.56
KIDMED score	6.63 ± 2.60
Physical activity	
Group A	N = 14
Group B	N = 30
Group C	N = 33

*BMI* body mass index, *WHR* waist/hip ratio, *TBW* total body water, *BMR* basal metabolic rate, *FFM* free fat mass, *FM* fat mass. Group A, physical inactivity; Group B, moderate-intensity physical activity; Group C, vigorous-intensity physical activity

### Genotyping

Genomic DNA was extracted from peripheral blood leukocytes using the Wizard Genomic DNA Purification Kit (Promega). The quantity and quality of DNA were assessed by using the NanoDrop spectrophotometer (NanoDrop™ One/OneC Microvolume UV–Vis Spectrophotometer; Thermo Fisher Scientific) and tested on agarose gel. NGS analysis was carried out using a targeted panel including genes and polymorphisms (see Additional file 1: Table S1), [16–29] related to diet, lifestyle, and physical performance/sports, on the Ion Gene Studio S5 platform (Thermo Fisher Scientific). The genes and specific SNPs were chosen for sequencing based on their significant biological roles in the lipid metabolic pathways and/or correlation with bioclinical parameters. The custom gene-panel was designed online using the Ion AmpliSeq™ Designer (https://ampliseq.com/browse.action) and resulted in 2-primer pools that are able to amplify 200 amplicons covering all the genes, with an amplicon range size of 125–275 bp. In particular, all the coding regions of genes were targeted including at least 25 bp of intronic flanking regions, and the promoter region of the following 9 out of 13 genes: *FNDC5, IL-6, CRP, HFE, TNF, IL1B, TGFB1, LPL, HMGCR*. The total size of the panel was 37.28 kb. Libraries were prepared using the Ion AmpliSeq™ Library Kit Plus following standard protocols and quantified using the Invitrogen™ Qubit™ Fluorometer to determine the dilution factor resulting in a concentration of ~ 100 pM. Balanced pooled libraries underwent template preparation and enrichment before being purified by using the Ion OneTouch™ ES (Thermo Fisher Scientific), according to the manufacturer’s protocol. The Ion Sphere Particles were loaded onto three different Ion 520 chips and sequenced with the Ion S5™ Sequencing Kit, using the Ion S5™ Sequencer (Thermo Fisher Scientific).

### Data analysis

Primary bioinformatic analysis (alignment against the GRCh37/hg19 human reference genome, quality and coverage analysis, and variant calling) was performed using the Torrent Suite™ Software 5.12 (Thermo Fisher Scientific), while the Variant Caller Parameter was set on ‘Germline–Low Stringency’ and annotated variants were filtered for genotype quality (GQ) < 5, flow space read depth (FDP) < 6, variant quality (QUAL) < 20, flow space alternate allele observations (FAO) < 2. The main coverage sequence was 30X. To compare our genotype frequencies, 1000GENOMES (www.1000genomes.org/1000-genomes-browsers) and GnomAD (gnomad.broadinstitute.org) databases were used, excluding the variants with very low frequencies (MAF < 1%), since the aim of our work was not to evaluate the pathogenic impact of rare variants.

### Linkage disequilibrium (LD) and haplotype analysis

Haploview (Barrett et al. 2005, www.broadinstitute.org/haploview) was used to determine the linkage disequilibrium (LD) between variants, to test the concordance of the genotype distribution with Hardy–Weinberg equilibrium and selecting representative SNPs of common variants (so-called tagSNPs) with MAF ≥ 0.05 by performing tagger analyses.

DnaSp v6 software was used for LPL haplotype reconstruction. The algorithm provided by PHASE [30] was used with 100 iterations, thinning intervals equal to 1, and 100 burn-in iterations [31].

### Association analysis

Association analysis by using single SNPs and haplotype with biochemical and body composition parameters was conducted using the general linear model (GLM) performed by Tassel 5.2.51v. For each marker-genotype combination, GLM finds the solution of ordinary least squares. The Tassel 5.2.51v software calculated the genotypic effect and not the allelic effect as deviations from the estimated value of the lower frequency genotype class. The class with the lowest frequency is set as zero effect, then the other genotype effects are given as deviations between their estimated values and the lowest frequency class [32]. Both the Fisher’s exact test and Chi-square test were used for evaluating significant pairwise associations and Bonferroni correction was also applied.

The effect of polymorphisms as well as of physical activity, WHR and KIDMED score on circulating levels of total cholesterol, LDL cholesterol, and triglycerides was investigated by calculating the explained variance (in percentage) of each of the biomarkers provided by each single polymorphism, WHR, and KIDMED score. Furthermore, to assess the independent effect of polymorphisms on total cholesterol, LDL cholesterol, and triglycerides, beyond and above the potential confounding effect of physical activity, WHR, KIDMED, and gender, multiple linear regression models were fitted. In these models, the strength of the relationship between each polymorphism and lipids was expressed as beta regression coefficients, and P values.

### eQTL analysis

Expression Quantitative Trait Loci (eQTL) analysis was performed using Genotype-Tissue Expression GTEx Portal v.8 (www.gtexportal.org) (eQTL), a data resource and tissue bank to study the relationship between genetic variants and gene expression in multiple human tissues and between individuals, for a better understanding of the genetic determinism of quantitative traits.

## Results

### NGS results

Targeted panel sequencing in 77 samples of healthy adolescents identified a total of 1964 variants. Two hundred and eight variants were found in the three genes of interest (*LPL, FNDC5, PPARγ*). Annotated variants were then filtered for quality parameters (FAO, FDP, GQ, QUAL) confirming the true positive calls. Of the 208 variants identified, 111 SNPs passed the quality filters (61 in *LPL*, 38 in *FNDC5* and 12 in *PPARγ*) (see Additional file 2: Table S2). Of these, 33 were insertions or deletions, 77 were single nucleotide variants and 1 substitution. The proportions of the 78 substitutions were: A/G, 14,1%; A/C, 7,5%; A/T, 5%; C/A, 8,7%; C/T, 16,6%; C/G, 5,1%; G/A, 14,1%; G/C, 2,6%; G/T, 6,4%; T/A, 2,6%; T/C; 16,6%; T/G, 2,6%.

Twenty-four of the total variants have not been previously described in the literature. In particular, 11 in *LPL* gene, 12 in *FNDC5,* and 1 in *PPARγ*. Among these, 21 SNV and 3 INDEL (see Additional file 2: Table S2). Of the 111 variants identified, 40 were located in the intronic region and 53 in the flanking regions (42 in 3'UTR, 4 in 5'UTR, 4 in upstream, 1 in an intergenic region, and 2 in a regulatory region). The remaining 18 variants were exonic (4 frameshift and 14 single substitutions) including one a stop codon variant. Of the 18 coding variants, three resulted in non-synonymous changes in *PPARγ*, from proline to alanine (P12A), from glycine to tryptophan (G290W), from lysine to glutamine (K156E), and two frameshift (L178Tfs*2; D7Rfs*5); three variants resulted in *FNDC5* of which one frameshift (L132Ffs*6) and two SNVs, leucine to valine (L81V) and histidine to asparagine (H127N); nine resulted in *LPL* including 4 synonymous variants, valine to valine (V135V), threonine to threonine (T388T), leucine to leucine (L81L), glutamate to glutamate (E145E); 4 non-synonymous changes, asparagine in serine (N318S), arginine to histidine (R197H), histidine to proline (H348P), aspartate in asparagine (E36N); one stop codon variant in exon 9 (S474X) and finally one frameshift (Q118Hfs*30).

Of the 24 novel variants that we identified, 5 had MAF ≥ 0.05, 2 had MAF between 0.05–0.01, and 17 had MAF < 0.01.

### Single SNP and multivariate analyses

**Fig. 1:**
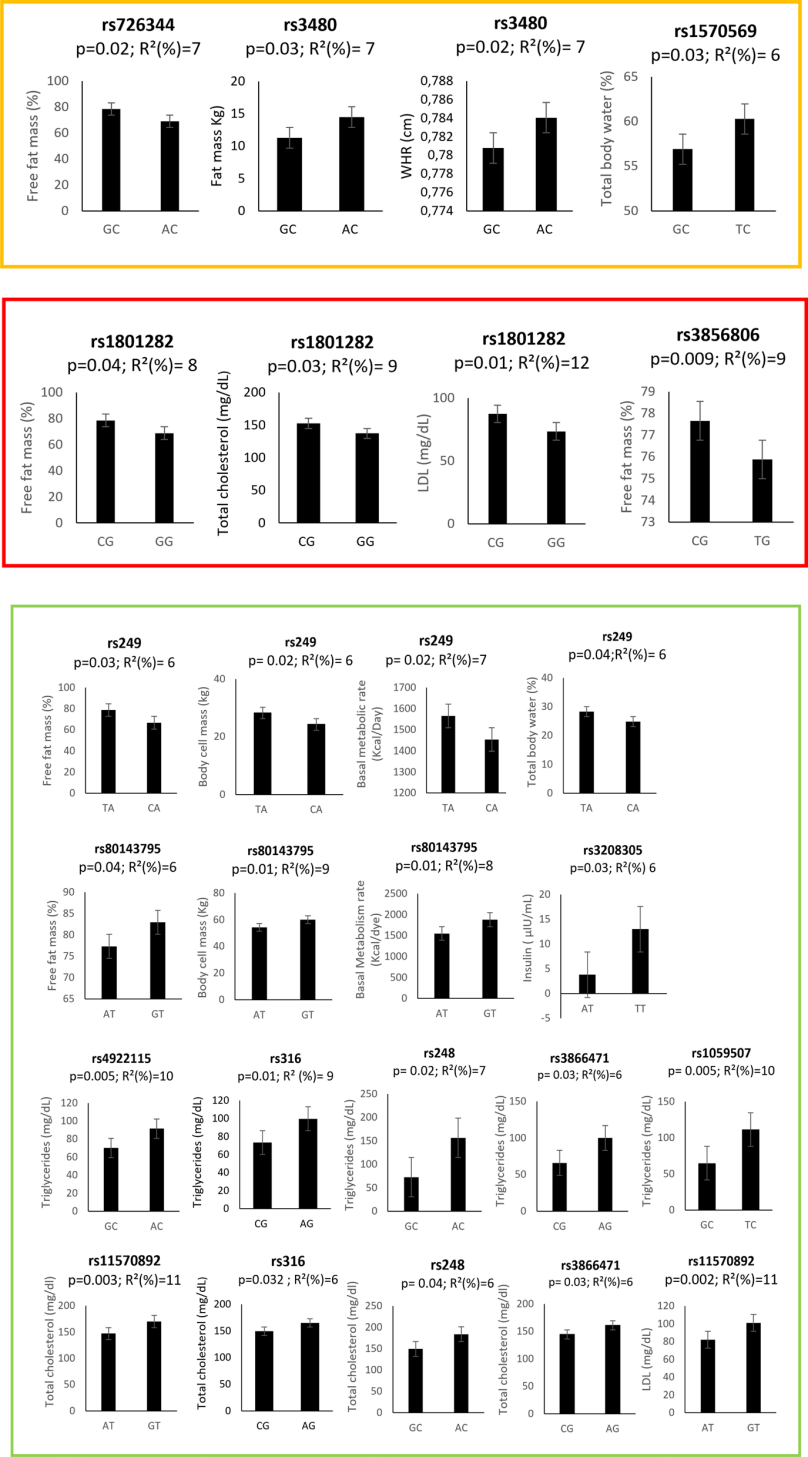
Significantly associated SNPs with different body composition parameters, lipid profile, and insulin levels in our cohort. The X-axis indicates the genotype status (letters) R^2^: is the statistical used for association analysis and p is the Benjamini–Hochberg Adjusted p-value. The associations of the polymorphisms in the three different genes are distinguished by squares of different colors: *FNDC5* in yellow, *PPARγ* in orange, and *LPL* in green

To identify tagSNPs in our cohort we used Haploview software v4.2. Fifty-two common SNPs were identified using R^2^ value of 0.8 of which 34, 19, and 3 in *LPL*, *FNDC5*, and *PPARγ* genes, respectively. For *FNDC5* and *PPARγ* no SNPs were in LD unlike some other SNPs in the LPL gene (rs11570892, rs4922115, rs1059507, and rs3866471). Subsequently, the relationship between these SNPs and either body composition assessment or lipid profile and insulin levels was investigated using the general linear model (GLM) by Tassel software, allowing to identify 58 significant associations (P < 0.01 and/or P < 0.05) after Bonferroni corrections. Among these, 26 related to parameters of our interest (WHR, TBW, FFM, FM, BCM, BMR, total cholesterol, LDL cholesterol, HDL cholesterol, triglycerides and insulin). Figure 1 shows significant associations between tag-SNPs and parameters related to lipid profile, anthropometric characteristics, and body composition.

**Fig. 2:**
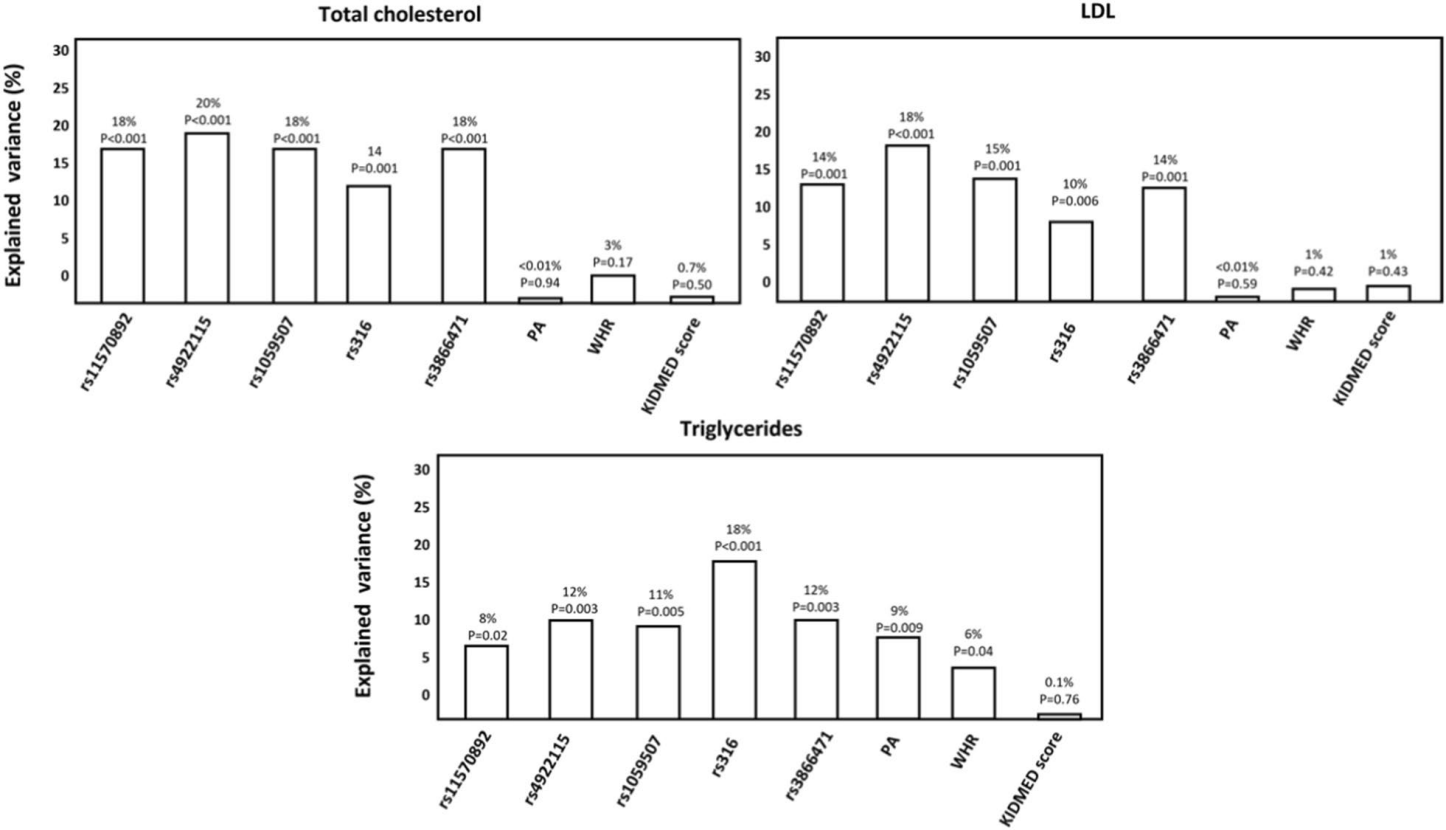
Lipid profile related to *LPL* polymorphisms and environmental factors. *PA* physical activity, *WHR* waist/hip ratio. Dependent (plasma lipid parameters) and independent (genotypes, PA, WHR, and KIDMED score) variables

Moreover, the effects of *LPL* polymorphisms as well as of physical activity, WHR and KIDMED score on circulating levels of total cholesterol, LDL cholesterol, and triglycerides are given in Fig. 2. As shown, the rs4922115 polymorphism was the first factor in rank explaining the total cholesterol and LDL cholesterol variability (rs4922115, rs11570892, rs1059507, and rs3866471 are in linkage disequilibrium, which explains the analysis result by GLM in which rs11570892 was the only one significantly associated with total cholesterol and LDL cholesterol levels) whereas the rs316 polymorphism resulted to be the first factor in rank explaining the variability of triglycerides (according to the ordinary linear regression analysis). Of note, physical activity, WHR, and KIDMED score largely failed to explain the variance of total cholesterol and LDL cholesterol (P = NS). Physical activity and WHR only explained the variability of triglycerides, but the strength of these associations was lower than that of polymorphisms.

It is worth noting that the genetic variants of *LPL* gene are located in 3’UTR with the exception of rs316. The analysis of variance for each SNP related to the lipid profile is shown in Table 2.

**Table 2:** *LPL* polymorphisms and analysis of variance related to blood lipid values

*snpID*	Variance explained (%)			p-value	Gene region	Location
	Cholesterol	LDL	Triglycerides			
rs11570892	18	14	8	< 0.001	*exon 10*	3'UTR
rs4922115	20	18	12	< 0.001	*exon 10*	3'UTR
rs3866471	18	14	12	< 0.001	*exon 10*	3'UTR
rs1059507	18	15	11	< 0.001	*exon 10*	3'UTR
rs316	14	10	18	< 0.001	*exon 8*	Exonic

Determining the grade of conservation of these variants by phastCons and phyloP methods [33], based on the multiple alignments of genome sequences of 46 different species, we observed that rs4922115 is the most conserved polymorphism, followed by rs11570892, rs3866471, while rs1059507 is the least conserved. Of note, data adjustment for confounders such as physical activity, KIDMED, WHR, BMI and gender did not affect the relationship between each polymorphism and serum lipids but for the links between rs11570892 and rs1059507 with serum triglycerides which just failed to reach the formal statistical significance (Table 3).

**Table 3:** Multiple linear regression models of lipids and *LPL* polymorphisms

snpID	Beta regression coefficient, and p values^a^		
	Cholesterol	LDL	Triglycerides
rs11570892	0.51 (< 0.001)	0.50 (< 0.001)	0.23 (0.067)
rs4922115	0.57 (< 0.001)	0.56 (< 0.001)	0.25 (0.038)
rs3866471	0.51 (< 0.001)	0.49 (< 0.001)	0.34 (0.004)
rs1059507	0.54 (< 0.001)	0.54 (< 0.001)	0.23 (0.060)
rs316	0.51 (< 0.001)	0.46 (< 0.001)	0.36 (0.003)

^a^Data were adjusted for physical activity, KIDMED, WHR, BMI and gender

### Haplotype analysis

**Fig. 3:**
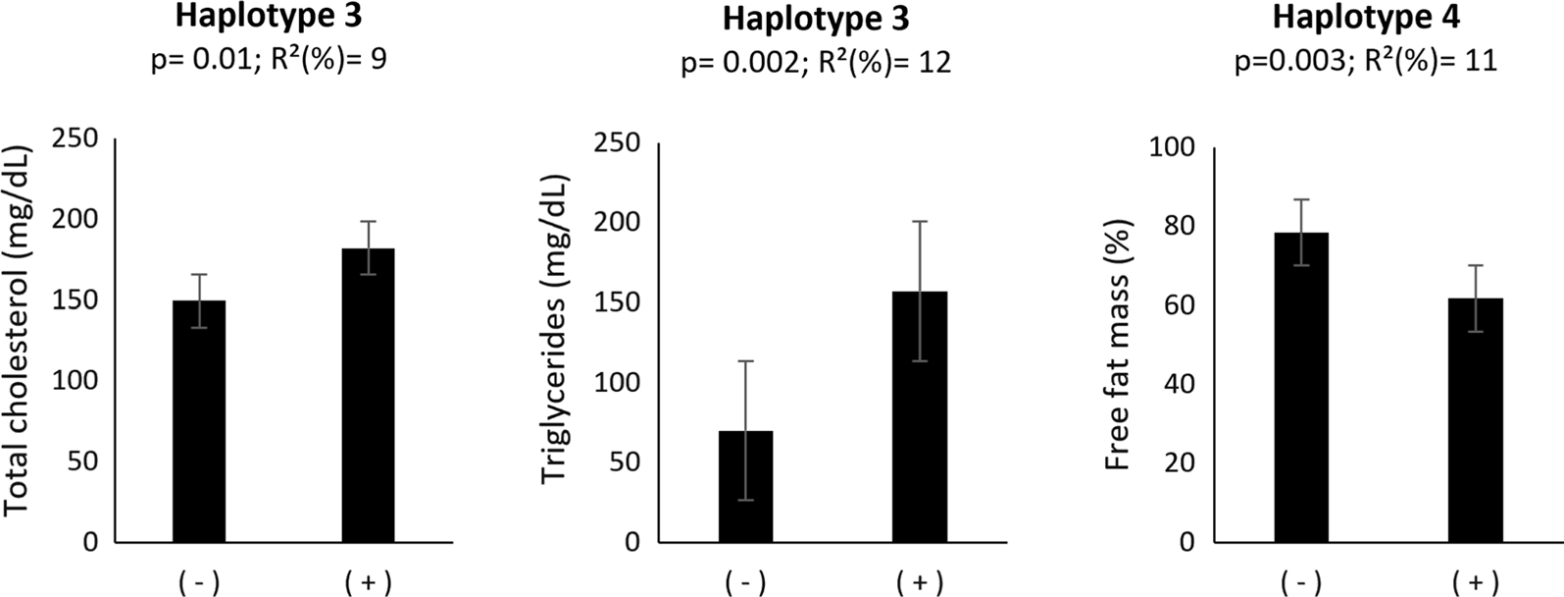
Significantly associated *LPL* haplotype with body composition and lipid parameters. The X-axis indicates the presence (+) or absence of the haplotype (−).*eQTLs*

Haplotype analyzes have been shown to be advantageous over single SNP analysis in the presence of multiple susceptibility alleles, particularly when SNPs are poorly correlated with each other [34]. Considering the small number of polymorphisms detected in two (*FNDC5* and *PPARγ*) of the three analyzed genes, we reconstructed the haplotypes using DnaSP logistic regression analysis for the *LPL* gene only. *LPL* haplotype reconstruction and analyses on significantly associated *LPL* polymorphisms (rs248, rs249, rs4922115, rs11570892, rs3208305, rs1059507, rs3866471) generated 27 different haplotypes. After excluding "very rare haplotypes" (haplotype detected in 1–2 samples out of 77) the blocks of haplotypes containing significant SNPs from the single SNP analysis to reduce multiple tests were tested for subsequent association analysis by the Tassel software using a GLM model. In particular, 9 distinct haplotypes were analyzed. GLM procedures identified associations between *LPL* haplotypes and parameters in our cohort, revealing 3 associations of blocks with lipid/body composition parameters, including: haplotype 3 (rs316, rs11570892, rs4922115, rs3866471, rs1059507, rs3208305), associated with increased levels of triglycerides (p = 0.002) and total cholesterol (p = 0.01); haplotype 4 (rs249, rs3208305), associated with the reduction of FFM (p = 0.003) (Fig. 3).

In order to understand the functional consequences of these genetic variations based on non-coding regions and how these affect quantitative traits, we evaluated the impact of genetic variations on gene expression in different human tissues, including the main users of fatty acids, through their eQTL mapping. Data retrieved from GTEx Portal v.8 showed a concordant tissue-specific effect for 5 *LPL* variants supporting their potential regulatory impact on the lipid parameters examined (Table 4).

**Table 4:** Variant tissue-specific effects by eQTLs analysis on LPL gene

SNPs	Alternative allele	p-value/NES					
		Thyroid	Adipose- Subcutaneous	Adipose Visceral (Omentum)	Whole blood	Kidney-cortex	Liver
**rs4922115**	A	**3.5e−5/0.25**	**4.2e−4/0.07**	0.08/0.05	1/2.4e**−**3	0.5/0.10	0.06/0.23
**rs11570892**	C	**8.7e−7/0.25**	**5.3e−4/0.07**	0.2/0.03	0.9/9.7e**−**3	0.5/0.10	0.06/0.23
**rs3208305**	T	**8.9e−7/0.21**	**1.3e−7/0.09**	**3.2e−3 /0.06**	**3.1e−7/0.22**	0.7/**− **0.06	**0.01/0.27**
**rs1059507**	T	**7.5e−7/0.26**	**3.1e−4/0.07**	0.08/0.05	1/2e**−**3	0.5/0.10	0.08/0.22
**rs3866471**	A	**3.7e−6/0.24**	**5.1e−4/0.07**	0.1/0.04	0.5/0.03	0.5/0.10	0.09/0.21

A p < 0.05 (marked in bold) was reported in the table indicating the existence of a relationship between a genetic variant and gene locus affecting gene expression in the analyzed tissue calculated by a T-statistics; NES indicates the normalized effect size across the tissues

## Discussion

In this study, to increase the understanding of the interaction between *LPL, PPARγ,* and *FNDC5* genes with lipid metabolic profile as well as body composition parameters, under non-pathological conditions, we have thoroughly evaluated the presence of novel and already discovered SNPs and their potential associations in young adolescents.

The role of many genetic variations in relation to lipid levels has already extensively been studied [12, 35, 36]. GWAS studies revealed that SNPs in the *LPL* are associated with levels of cholesterol, triglycerides, and more, as well as numerous molecular phenotypes are related to human diseases and diseases risk [6, 37, 38]. Furthermore, controversial data have been reported on the association (positive, null, or negative) between irisin levels and BMI, body weight and FM in different populations [39–41] and different associations between lipid intake and BMI in carriers and non-carriers of *PPARγ* polymorphisms have been observed [42].

In our cohort, we identified 111 variants, of which 24 were novel. All genotyped variants were in concordance with Hardy–Weinberg equilibrium expectations. The single SNP analysis, performed by linear regression, revealed 26 significant associations (p < 0.05) of which 10 with body composition parameters and 16 with lipid profiles. The best associated *PPARγ* SNP with FFM was rs3856806 (p = 0.009) while *FNDC5* SNPs significantly associated with FFM (rs726344, p = 0.02), FM and WHR (rs3480, p = 0.02; p = 0.03 respectively), and TBW (rs1570569, p = 0.03). A significant association, never previously described, was identified between the intronic variant c.775 + 80A > G in *LPL*, rs80143795, and FFM, BCM, and BMR.

In this study, the *LPL* haplotype association results, largely comparable with the single-site analyses, revealed two main blocks (3 and 4) significantly associated with lipid and body composition parameters never reported before. In addition, considering the explained variance effects of *LPL* polymorphisms, physical activity, WHR, and KIDMED score on lipid profile, we discovered that rs316 and rs4922115 were the main factors explaining the variability of triglycerides and total/LDL cholesterol, respectively. The rs316 is a synonymous variant located in exon 8 not in linkage disequilibrium with other identified SNPs, previously described to be associated with HDL cholesterol in a healthy African Black population [43]. On the other hand, the individual effect of the non-coding rs4922115 on blood lipids has not been previously investigated in other populations except in Asian Indians [44]. However, this study did not report any statistically significant interaction between this SNP and lipid parameters. In our study rs4922115, such as rs316, was part of haplotype block 3 and was in LD with rs11570892, rs1059507, and rs3866471 (R^2^ = 0.58–0.67). In particular, most of these SNPs are located in the 3’UTR of the gene known to be involved in gene regulation, affecting LPL mRNA levels, or post-transcriptional changes [45].

Several genetic polymorphisms in the 3′-UTR of different genes influencing gene expression, have already been reported in humans relating to various disorders. For example, rs1059611, significantly associated with increased HDL and decreased TG concentrations in patients with coronary heart disease, affects mRNA stability and LPL expression. Specifically, in homozygous and heterozygous states, the C allele carriers show increased gene expression [46]. In addition, a functional study of rs6773957, strongly associated with plasma adiponectin levels and body weight in patients affected by metabolic syndrome, indicates that this variant may affect mRNA stability influencing the expression of ADIPOQ [47]. Very often, 3’UTR SNPs influence gene expression by secondary structures, in the form of stem-loops on the mRNA [48] such as rs27770 of *Polo-like Kinase 1* (*PLK1*) which, showing variations in secondary mRNA structures and stability, leads to increased gene expression [49].

Different studies have been strongly associated these 3’UTR-SNPs with HDL cholesterol and TG levels, especially in Europeans [50, 51]. Moreover, it has been previously shown that haplotypes in these regions modulate pathologies such as atherosclerosis, insulin resistance, blood pressure, and obesity [52].

Interestingly, the study of genetic variations identified in the LPL gene on the molecular and/or phenotypic consequences through genome-wide mapping of the eQTL analysis showed the increased expression of rs4922115 and other 4 unique eQTL SNPs (rs11570892, rs3208305, rs1059507, and rs3866471) in thyroid and adipose subcutaneous tissues, supporting the impact of these variants on the lipid parameters examined (Table 4). Among all these eQTL variants, rs3208305, found to be significantly associated with insulin levels in this study, showed a very strong deregulated expression also in other tissues such as adipose visceral and whole blood. However, determining causal variants, molecular mechanisms and relevant tissues contributing to these associations can be difficult, largely due to the ability of numerous tissues to synthesize and metabolize fatty acids [53]. Furthermore, the concordant deregulated expression of *LPL* gene SNPs in the thyroid tissue should not surprise considering the contribution of thyroid hormones to energy expenditure, heat production, and direct regulation of basal metabolism [54].

Overall, despite recent studies suggest that a high-quality diet affects well-being [55], a "healthy" lifestyle is not enough to prevent potential conditions “at-risk”. In addition to physical activity and muscle mass, other factors, such as hormonal structure, temperature, and genetics, influence basal metabolism [56]. Our results showed that SNPs constituting the haplotypes were significantly associated with different lipid parameters (triglycerides, total cholesterol, and LDL cholesterol), while lifestyle habits, such as adherence to MD and physical activity, as well as WHR did have a low or null impact in healthy adolescents, highlighting the important role of genetic variants.

It is worth noting that the importance of diet and exercise in preventing cardiovascular disease among healthy young adults has been recently confirmed [57, 58]. Therefore, large-scale longitudinal studies evaluating the outcome among these young adults will be helpful for better identification and modification of risk factors in certain high-risk haplotypes.

Several variables influencing the interpretation of data in our cohort and/or explaining different results from previous studies include the use of sampling criteria (e.g. affected population/healthy population; inclusion of very young subjects compared to those predominantly selected, ethnicity, etc.) different software tools and algorithms applied for variant analysis, although the major limitation of this study is the sample size used for association analyses.

Overall, this cohort study of young healthy individuals is of significant importance since it will add new knowledge to the role of genetic factors in maintaining health status. In fact, cataloguing of DNA polymorphisms in different populations is critical to the development of genome-based knowledge about an individual's susceptibility to many common diseases and, at the same time, will be essential to produce a safer and more effective individualized diet.

In future, it is hoped that research will unveil ways to make SNP useful markers for clinical setting. Finding out how SNPs affect the health of an individual and then transforming this knowledge into the development of new drugs will undoubtedly revolutionize the treatments of the most common devastating disorders.

## Conclusions

To the best of our knowledge, this study is the first investigation reporting the analysis of sequence variations in the *LPL, PPARγ, and FNDC5* genes and their associations with anthropometric/body composition and lipid traits, in young healthy individuals, who adhere to the MD and perform physical activity.

While our results support, even in a young cohort, previously reported data on the association of non-coding SNPs and lipid parameters, on the other hand, it emerges the fundamental contribution in these associations of the genetic component with respect to the environmental factors.

However, despite the large number of significant associations between SNPs and phenotypes, most associations have not yet been replicated, which is why further studies are needed to better elucidate the role of these variants in affecting interindividual variation in plasma lipid phenotypes, leading to improvements in quality of life through better preventive strategies.

## Supplementary Information

**Additional file 1:** **Table S1**. Diet, lifestyle, and physical performance/sports - related genes examined in this study.

**Additional file 2** **Table S2**. Annotated variants detected by targeted NGS analysis in our cohort.

## Data Availability

The data presented in this study are available in results and additional files sections.

## Abbreviations

SNPs: Single nucleotide polymorphisms
MD: Mediterranean diet
LPL: Lipoprotein lipase
FNDC5: Fibronectin type III domain containing protein 5
PPARγ: Peroxisome proliferator-activated receptor gamma
PCR: Polymerase chain reaction
NGS: Next generation sequencing
DIMENU: Dieta Mediterranea and Nuoto
BMI: Body mass index
WHR: Waist/hip ratio
TBW: Total body water
BMR: Basal metabolic rate
FFM: Free fat mass
FM: Fat mass
GQ: Genotype quality
FDP: Flow space read depth
QUAL: Variant quality
FAO: Flow space alternate allele observations
MAF: Minor allele frequency
GLM: General linear model
eQTL: Expression quantitative trait loci
LD: Linkage disequilibrium
GWAS: Genome-wide association study
