# Supplementary material for: LPL, FNDC5 and PPARγ gene polymorphisms related to body composition parameters and lipid metabolic profile in adolescents from Southern Italy
Source: J Transl Med. 2022 Mar 3;20:107. doi: 10.1186/s12967-022-03314-w (PMC8895817; doi:10.1186/s12967-022-03314-w)
Supplement: Supplementary file 1 — Additional file 1: Table S1. Diet, lifestyle, and physical performance/sports - related genes examined in this study. [file 12967_2022_3314_MOESM1_ESM.pdf]

Gene	Chromosomal location	Biological process	References
FNDC5	1p35.1	Homeostatic metabolism	[16]
IL-6	7p15.3	Inflammation and the maturation of B cells	[17]
CRP	1q23.2	Host defense	[18]
HFE	6p22.2	Intestinal absorption of iron	[19]
PPARG	3p25.2	Nuclear receptor involved in physical performance	[20]
IL-10	1q32.1	Immune regulatory cytokine that effects in inflammation	[21]
TNF	6p21.33	Cell proliferation, differentiation, apoptosis, lipid metabolism and coagulation	[22]
IL1B	2q14.1	Prostaglandin synthesis, fibroblast proliferation, collagen production and differentiation of T-cells	[23]
TGFB1	19q13.2	Cell proliferation, differentiation and growth, expression and activation of other growth factors	[24]
LPL	8p21.3	Triglyceride metabolism, lipid clearance and cholesterol metabolism	[25]
HMGCR	5q13.3	Cholesterol biosynthesis, cholesterol metabolism, lipid clearance	[26]
CETP	16q13	Transfer of neutral lipids, including cholesteryl ester and triglyceride, among lipoprotein particles	[27]
LIPC	15q21.3	Hydrolysis of triglycerides and phospholipids present in circulating plasma lipoproteins	[28]
FADS1/2	11q12.2	Essential fatty acid metabolism	[29]

Supplemental Table S1. Diet, lifestyle, and physical performance/sports - related genes examined in this study.
