# Supplementary material for: LPL, FNDC5 and PPARγ gene polymorphisms related to body composition parameters and lipid metabolic profile in adolescents from Southern Italy
Source: J Transl Med. 2022 Mar 3;20:107. doi: 10.1186/s12967-022-03314-w (PMC8895817; doi:10.1186/s12967-022-03314-w)
Supplement: Supplementary file 2 — Additional file 2 Table S2. Annotated variants detected by targeted NGS analysis in our cohort. [file 12967_2022_3314_MOESM2_ESM.pdf]

Gene	Variation	Amminoacid change	Gene region	RefSNP ID	Chr. Position	Location	Alleles	Type	Variant Effect	MAF
LPL	c.*1142C>T		exon 10	rs1059507	19823963	3'UTR	C/T	SNV	unknown	0.126
	c.*1742T>C			rs1059611	19824563	3'UTR	T/C	SNV	unknown	0.21
	c.*1806_*1807insTT		exon 10	rs10645926	19824626	3'UTR	/ TT	INDEL	unknown	0.1013
	c.405G>A	p.Val135=	exon 3	rs1121923	19809435	exonic	G/A	SNV	synonymous	0.049
	c.1428-11C>T		intron 9	rs11570891	19822810	intronic	C/T	SNV	unknown	0.107
	c.*796A>G		exon 10	rs11570892	19823617	3'UTR	A/G	SNV	unknown	0.168
	c.*1671T>C		exon 10	rs13702	19824492	3'UTR	T/A	SNV	unknown	0.49
	c.*1846C>T		exon 10	rs1446407392	19824667	3'UTR	C/T	SNV	unknown	0.33
	c.430-47A>C		intron 3	rs1554517533	19953263	intronic	A/C	SNV	unknown	0.00000895
	**c.*853C>G**		**exon 10**	**rs1803924**	**19823674**	**3'UTR**	**C/T**	**SNV**	**unknown**	**0.101**
	c.541+74T>C		intron 4	rs249	19953495	intronic	T/C	SNV	unknown	0.0791
	c.775+33C>G		intron 5	rs254	19811897	intronic	C/G	SNV	unknown	0.146
	c.775+37T>C		intron 5	rs255	19811901	intronic	T/C	SNV	unknown	0.146
	c.775+103C>T			rs256	19811967	intronic	C/T	SNV	unknown	0.138
	c.953A>G	p.Asn318Ser	exon 6	rs268	19956018	exonic	A/G	SNV	missense	0.039
	c.1139+43T>C		intron 7	rs301	19816934	intronic	T/C	SNV	unknown	0.241
	c.1164C>A	p.Thr388=	exon 8	rs316	19960925	exonic	C/A	SNV	synonymous	0.039
	c.*1250A>G		exon 10	rs3200218	19824071	3'UTR	A/G	SNV	unknown	0.252
	c.69_70insC	p.Gly24ArgfsTer17	exon 1		19797020	3'UTR	/ C	INDEL	frameshiftInsertion	**0.23**
	c.*827A>T			rs3208305	19823648	3'UTR	A/T	SNV	unknown	0.281
	c.1421C>G	p.Ser474Ter	exon 9	rs328	19819724	exonic	C/G	SNV	nonsense	0.101
	c.1427+356A>G		intron 9	rs329	19962575	intronic	A/G	SNV	unknown	0.12
	c.430-34C>A		intron 3	rs343	19953276	intronic	C/A	SNV	unknown	0.20
	c.*1224C>A			rs3735964	19824045	3'UTR	C/A	SNV	unknown	0.101
	**c.*1848C>A**		**exon 10**	**rs3866471**	**19824669**	**3'UTR**	**C/A**	**SNV**	**unknown**	**0.162**
	c.*9G>A			rs4922115	19822830	3'UTR	G/A	SNV	unknown	0.12
	c.*1895T>TA				19824716	3'UTR	/ A	INDEL	unknown	<0.01
	c.249+22_249+23insG				19805873	intronic	/ G	INDEL	unknown	<0.01
	c.*371T>C		exon 10	rs3289	19823192	3'UTR	T/C	SNV	unknown	0.039
	**c.590G>T**	**p.Arg197Leu**	**exon 5**	**rs372668179**	**19954168**	**exonic**	**G/A**	**SNV**	**missense**	**0.001**
	**c.*1387T>C**		**exon 10**	**rs1803923**	**19966697**	**3'UTR**	**T/C**	**SNV**	**unknown**	**0.003**
	**c.775+80A>G**		**intron 5**	**rs80143795**	**19954433**	**intronic**	**A/G**	**SNV**	**unknown**	**0.0000648**
	c.541+97G>A		intron 4	rs1160372282	19953518	intronic	G/A	SNV	unknown	<0.01
	c.-283G>T			rs80351041	19939158	intronic	G/T	SNV	unknown	0.03
	c.430-58C>A		intron 3	rs1043637503	19953252	intronic	C/A	SNV	unknown	<0.01

	c.776-7del		intron 5	rs763904323	19813339	intronic		INDEL	unknown	<0.01
	**c.*717C>T**		**exon 10**	**rs1427611839**	**19966027**	**3'UTR**	**C/T**	**SNV**	**unknown**	**<0.01**
	**c.1322+34A>T**		**intron 8**	**rs1563581796**	**19961117**	**intronic**	**A/T**	**SNV**	**unknown**	**<0.01**
	c.1140-13C>T		intron 7	rs756414875	19960888	intronic	C/T	SNV	unknown	0.000008798
	c.*172C>A		exon 10	rs1200136169	19965482	3'UTR	C/A	SNV	unknown	0.001
	c.*828A>C		exon 10	rs1243571209	19966138	3'UTR	A/C	SNV	unknown	<0.01
	c.1043A>C	p.His348Pro	exon 7	rs1563579671	19959284	exonic	A/C	SNV	missense	<0.01
	c.1427+78A>C		intron 9	rs1590148879	19962297	intronic	A/C	SNV	unknown	<0.01
	c.*820A>G		exon 10		19823641	3'UTR	A/G	SNV	unknown	<0.01
	c.-281T>G		exon 1	rs1800590	19939160	upstream	T/G	SNV	unknown	0.47
	c.106G>A	p.Asp36Asn		rs1801177	19948197	exonic	G/A	SNV	missense	0.013
	c.249+82_249+83insC		intron 2	chr8:19805933	19805934	intronic	/ C	INDEL	unknown	<0.01
	c.*174_*176delCTAinsACT		exon 10	chr8:19822995	19822995	3'UTR		Substitution	unknown	<0.01
	c.*1127G>T		exon 10	19823948	19823948	3'UTR	G/T	SNV	unknown	<0.01
	c.*1916_*1917insA		exon 10	chr8:19824737	19824738	3'UTR	/ A	INDEL	unknown	<0.01
	**c.249+7G>C**		**intron 2**	**rs1161286800**	**19948347**	**intronic**	**G/C**	**SNV**	**unknown**	**0.0001**
	**c.541+97_541+98insTG**		**intron 4**	**rs1160372282**	**19811029**	**intronic**		**INDEL**	**unknown**	**<0.01**
	c.-113A>C		exon 1	rs771569574	19939328	5'utr	A/C	SNV	unknown	<0.01
	c.*547T>C		exon 10	rs995558109	19965857	3'UTR	T/C	SNV	unknown	<0.01
	c.430-34C>A		intron 3	rs343	19810787	intronic	C/A	SNV	unknown	0.20
	**c.353A>G**	**p.Gln118HisfsTer30**	**exon 3**	**rs1176046872**	**19809383**	**exonic**	**A/C**	**SNV**	**frameshiftInsertion**	**0.001**
	c.*1742T>C		exon 10	rs1059611	19824563	3'UTR	T/C	SNV	unknown	0.21
	**c.*795C>T**		**exon 10**	**rs1314699988**	**19823616**	**3'UTR**	**C/T**	**INDEL**	**unknown**	**<0.01**
	c.*1805C>CTT		exon 10	rs10645926	19824626	3'UTR	/ TT	INDEL	unknown	0.286
	c.435G>A	p.Glu145=	exon 4	rs248	19953315	exonic	G/A	SNV	synonymous	0.013
	c.775+80A>G		intron 5	rs80143795	19954433	intronic	A/G	SNV	unknown	0.13
FNDC5	c.275-34T>C		intron 4	rs1284368	33330432	intronic	A/G	SNV	unknown	0.016
				rs1298190	33340038	upstream	G/A	SNV	unknown	<0.01
	**c.164A>C**		**intron 5**	**rs1338123015**	**32864446**	**intronic**	**T/G**	**SNV**	**unknown**	**<0.01**
	c.-127-450_-127-445del		intron 1	rs1361479925	33335040	intronic		INDEL	unknown	0.07
	c.1800_1801dup		exon 5	rs138570533	33328409	3'UTR	G/GGA	INDEL	unknown	0.06
	c.241C>T	p.Leu81=	exon 4	rs138727728	32867786	exonic	G/A	SNV	synonymous	0.01
				rs1448537936	32872520-32872526	regulatory region intron	TCTTTCT/TCT	INDEL	unknown	<0.01
	c.-128+1039C>G		intron 1	rs1570569	33336956	intronic	G/C	SNV	unknown	0.14
	c.2046C>T			rs3480	33328165	3'UTR	G/A	SNV	unknown	0.416
				**rs57219418**	**32874343**	**intergenic**	**C/T**	**SNV**	**unknown**	**0.29**

	c.274+250C>T		intron 4	rs726344	33333104	intronic	G/A	SNV	unknown	0.147
	c.-127-446G>C		intron 1	rs142773183	32869439	intronic	C/G	SNV	unknown	0.04
	c.-4135A>G				33338109	upstream	T/C	SNV	unknown	<0.01
	c.-4081T>A				33338055	5'UTR	A/T	SNV	unknown	<0.01
	**c.677G>A**			**rs1043065315**	**33329534**	**3'UTR**	**C/T**	**SNV**	**unknown**	**<0.01**
	**c.1229C>T**			**rs41265869**	**32863381**	**3'UTR**	**G/A**	**SNV**	**unknown**	**0.05**
	**c.274+326C>A**		**intron 4**	**rs974722844**	**32867427**	**intronic**	**G/T**	**SNV**	**unknown**	**<0.01**
				rs910923450	32872490-32872494	regulatory region intron	TC/	INDEL	unknown	<0.01
	c.274+85A>G		intron 4	rs1187456844	32867668	intronic	T/C	SNV	unknown	<0.01
	c.1055_1057del			rs1243458159	33329156	3'UTR	TTA/	INDEL	unknown	<0.01
	**c.393delG**	**p.Leu132PhefsTer6**	**exon 5**	**rs1164445439**	**32864679**	**exonic**	**C/ T**	**INDEL**	**frameshiftDeletion**	**<0.01**
	**c.1829G>A**			**rs1429301233**	**32862781**	**3'UTR**	**C/T**	**SNV**	**splice region variant**	**<0.01**
	c.346dup	p.Thr116AsnfsTer90		rs1245625486	32864726-32864731	intronic	/ T	INDEL	frameshift/intron	<0.01
	c.157A>G			rs1403315264	32864453	3'UTR	T/C	SNV	unknown	<0.01
	c.1973_1974insC				33328238	3'UTR	/ C	INDEL	unknown	<0.01
	c.1389delA				33328822	3'UTR	A /	INDEL	unknown	<0.01
	c.274+325T>A		intron 4	rs1025936314	32867428	intronic	A/T	SNV	unknown	<0.01
	c.*643_*644insA		exon 5		33329568	3'UTR	/ T	INDEL	unknown	<0.01
	**c.*825T>C**			**rs191148874**	**32863785**	**3'UTR**	**A/G**	**SNV**	**unknown**	**0.02**
	**c.379C>A**	**p.His127Asn**	**exon 5**	**rs759315666**	**32864693**	**exonic**	**G/T**	**SNV**		**<0.01**
	c.*1078dup				33329133	3'UTR	/ T	INDEL	unknown	<0.01
	c.-208_-202del			rs1029941501	32872468	5'utr	TCTCTTT (7bp) /	INDEL	unknown	<0.01
					33338137	upstream	/ C	INDEL	unknown	<0.01
	c.*1348G>A			rs188449869	32863262	3'UTR	C/T	SNV	unknown	0.01
	**c.*1059A>T**			**rs559443224**	**33329152**	**3'UTR**	**T/A**	**SNV**	**unknown**	**0.006**
	c.275-34T>C			rs1284368	33330432	intronic	A/G	SNV	unknown	0.016
	c.274+23T>C		intron 4	rs1258376954	33333330	intronic	A/G	INDEL	unknown	<0,01
	**c.-127-446G>A**			**rs142773183**	**33335040**	**intronic**	**C/T**	**SNV**	**unknown**	**0.07**
PPARγ	c.34C>G	p.Pro12Ala	exon 1	rs1801282	12393125	exonic	C/G	SNV	missense	0.124
	c.1431C>T	p.His477=	exon 7	rs3856806	12475557	3'UTR	C/T	SNV	synonymous	0.126
	c.531_532insA	p.Leu178ThrfsTer2	exon 4	rs1469268585	12434163	exonic	T/TA	INDEL	frameshiftInsertion	0.00000797
	c.481-13dupT				12434094	intronic	C/CT	INDEL	unknown	<0.01
	c.868G>T	p.Gly290Trp	exon 5		12447629	exonic	G/T	SNV	unknown	<0.01
	c.39del	p.Val14Ter	exon 5	rs587776687	12475349	intronic	A/AT	INDEL	nonsense	<0.01

	c.-79dupT				12393009	5'_utr	/ T	INDEL	unknown	<0.01
	c.850dup	p.Glu284GlyfsTer68	exon 5	rs769209078	12475349	intronic	/ G	INDEL	frameshift	<0.01
	**c.550A>G**	**p.Lys156Glu**	**exon 4**	**rs1575106051**	**12392683**	**exonic**	**A/G**	**SNV**	**synonymous**	**<0.01**
	c.310+16dup		intron 2		12421443	intronic	/ T	INDEL	unknown	<0.01
	c.18_19insC	p.Asp7ArgfsTer5	exon 1		12393109	exonic	/ C	INDEL	frameshiftInsertion	<0.01
	c.874_875del	p.Leu292ValfsTer59	exon 5	rs1287260681	12406133-12406137	non coding	TT/	INDEL	non coding transcript	< 0.01

Additional file Table S2. List of variants identified in this study.
